# Physiochemical, sensorial, and rheological characteristics of sauce developed from Kashmiri apples: Influence of cultivars and storage conditions

**DOI:** 10.1002/fsn3.2239

**Published:** 2022-05-07

**Authors:** Rayees Ahmad Bakshi, Afra Aslam, Zakir S. Khan, Shumaila Fayaz, B.N. Dar

**Affiliations:** ^1^ Department of Food Science & Technology University of Kashmir Srinagar Jammu and Kashmir India; ^2^ Department of Food Technology Islamic University of Science and Technology Pulwama Jammu and Kashmir India

**Keywords:** apples, processing, rheology, sauce, sensory evaluation

## Abstract

The present investigation was undertaken to develop sauce from different cultivars of apples. Apple sauce of 5 cultivars was developed and effect of the storage conditions on the pH, acidity, TSS, total sugar, color, sensory, and rheological behavior of different apple sauce cultivars was studied. Analytical determinations were made after 0, 15, 30, and 45 days at both refrigerated and ambient conditions. The observed range of TSS was 30 to 30.14˚brix for refrigerated and 27.4 to 30.7˚brix for sauces stored at ambient storage conditions.. The pH decreased during the overall storage period from 4.07 to 3.96 in refrigerated samples, while as pH decreased from 4.06 to 3.92 in ambient stored samples. Rheological properties of sauces were evaluated using a parallel plate rheometer that showed the storage modulus, Gʹ higher than the loss modulus, G″ for all the sauce samples indicating the dominance of the elastic behavior. The viscosity decreased with an increase in the shear rate for both, refrigerated and ambient stored sauce samples at the end of the storage period. Organoleptic characteristics (taste, color, aroma, and appearance) were examined by a semi‐trained panelist using 5 point hedonic scale. The sauce samples from Mollies Delicious and Chamure apple cultivars showed the highest acceptance.

## Graphical abstract

Apple is considered as fourth important fruit throughout the world (Zarein et al., 2015);however, the shelf life of apple is limited and decline in quality is rapid during storage (Vieira et al., 2009). Loss reduction can be achieved by converting apple into apple products like sauces, especially for those countries, which lack efficient post‐harvest system . Physiochemical properties, color, sensory, and rheology are very important in the formulation of fruit‐related products for the technical, customer acceptibility and marketing reasons. These all parameters together can be useful for optimal design of unit operations required to formulate and handle the product like sauces, from processing to consumption.

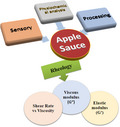

## 1 INTRODUCTION

In India, Jammu and Kashmir is the main apple producing state and produces around 60% of the apples of the country. India lacks efficient post‐harvest systems, which lead to the loss of around 30% agricultural and horticultural produce, therefore, conversion to related processed products can be the best option to minimize the losses (Bhardwaj & Pandey, 2011). Apple is considered as 4th important fruit throughout the world (Zarein et al., 2015). Apple (*Malus domestica*) is consumed throughout the world as it contains vitamins, dietary fiber, and bioactive compounds (phenolic compounds) besides carbohydrates, proteins, and lipids (Sun et al., 2002). Consumption of apple is usually in natural form; however, the service life of apple is limited and decline in quality attributes is rapid during storage (Vieira et al., 2009). Preservation of apples for optimum periods can be achieved by processing apple into different processed products, like jams, jellies, and even sauces (Gould, 2000). Processed products like fruit sauces are considered a big turn in the food industry and are an important part of the infant diet. These processed products are a valuable source of micronutrients, antioxidants, and fiber fostering health benefits for infants (Patras et al., 2009). There is an increase in the demand for apple sauce throughout the world by around 13% and more than 13% in Asia and the Middle East (Parker, 2011). Apple sauce is mainly used as snacks, it can also be used as a functional alternative to chocolate. It is also used as between‐meal food particularly by children (Colin‐Henrion et al., 2009). Besides being a rich source of nutrients, these sauces must be acceptable to the consumers from the sensory point of view. Nowadays, consumers are apprehensive about the quality attributes of processed food product; therefore, it is necessary to have an understanding of physiochemical changes and rheological parameters as processing (cooking) and storage can modify the characteristic parameters of the processed product (Nindo et al., 2007). Furthermore, there is limited literature available on apple sauce developed from indigenous cultivars of apple. Therefore, considering the above facts the objectives of the research are to study the physicochemical and rheological properties of five different Kashmiri cultivars of apple sauce during the storage period of 45 days at ambient and refrigerated temperatures.

## 2 MATERIALS AND METHODS

### 2.1 Procurement of raw material

Fresh, ripe, and sound fruits of 5 apple cultivars American Apirouge, Delicious, Red Delicious, Mollies Delicious, Chamure, devoid of any microbial infection or mechanical fissures were procured from the local market of Srinagar and then bought to the food processing and training center of Islamic University of Science and Technology, Awantipora for further processing.

### 2.2 Preparation of samples

Apple processing was done according to Wani et al., (2009). Apples were first sorted and damaged fruits were discarded. The selected apples were washed, peeled, and followed by the removal of core and seeds. The pulp was obtained by fruit pulper, strained, and cooked with 1/3rd amount of sugar followed by spice addition (bag method) to the desired consistency (Wani et al., 2009). The standard recipe used for apple sauce making was as follows: apple pulp 1 kg, sugar 250 g, salt 10 g, onion (chopped) 200 g, ginger (chopped) 100 g, garlic (chopped) 50 g, red chili powder10 g, clove (headless) 5 numbers, cinnamon, cardamom (large), aniseed (powdered) 15 g each, vinegar/ acetic acid 50 ml, and sodium benzoate or KMS 0.7 g per kg sauce. The final product was filled hot in sterilized bottles stored and divided into ten groups (T0, T1, A0, A1, B0, B1, C0, C1, D0, and D1) with each cultivar consisting of two groups for both refrigerated and ambient storage study as shown in Table 1.

**TABLE 1:** Treatment formulation

S. No.	Apple cultivar	Refrigerated storage	Ambient storage
		Treatments	
1	American Apirouge	T_0_	T_1_
2	Delicious	A_0_	A_1_
3	Red Delicious	B_0_	B_1_
4	Mollies Delicious	C_0_	C_1_
5	Chamure	D_0_	D_1_

### 2.3 Physicochemical analysis of the sauce

#### 2.3.1 Determination of pH

The pH of the apple pulp was determined using a digital pH meter (the pH meter, mod. Cyberscan 510). The calibration of the pH meter was done before sample analysis, then the required amount of sample was taken and brought into contact with the electrode of the pH meter, and reading was noted.

#### 2.3.2 Determination of titratable acidity

The method used for calculating *titratable* acidity was done as per the procedure of (Horwitz, 2000). The percentage of *titratable* acidity was calculated as below.

$$\mathrm{Acidty}\%\left(g/L\right)=\frac{\mathrm{Titre}\mathrm{value}\times \mathrm{Normality}\mathrm{of}\mathrm{alkali}\times \mathrm{Vol}\mathrm{made}\mathrm{up}\times \mathrm{Equivalent}\mathrm{wt}.\mathrm{of}\mathrm{acid}\times 100}{\mathrm{volume}\mathrm{of}\mathrm{Sample}\mathrm{taken}\mathrm{for}\mathrm{estimation}\times \mathrm{wt}\mathrm{or}\mathrm{vol}.\mathrm{of}\mathrm{sample}\times 1000\times 100}$$

#### 2.3.3 Determination of total solid soluble solids and total solids

Calibrated refractometer (Atago) was used for estimation of total soluble solids and total solids in Brix according to (Wani et al., 2009).

#### 2.3.4 Determination of color

A hunter laboratory color spectrophotometer (Colorflex, Hunterlab) was used to measure the L, a* and b* values of the four samples. The colorimeter was calibrated using standard white and blackboards. The color values were expressed as L (whiteness to darkness), a (redness to greenness), and b (yellowness to blueness). The samples were placed in a transparent petri dish covered with a standard black cup and placed against the light source for color measurement. For each sample, three measurements were taken at different positions of the sample.

#### 2.3.5 Determination of rheological characteristics

The rheological properties were studied using Rheometer Physica MCR 101 (Anton Paar). The parallel plate geometry with 0.5 mm gap was used, and tests were conducted at a constant temperature of 25°C. To evaluate visco‐elastic characteristics (loss modulus, Gʺ, and storage modulus, Gʹ), the dynamic oscillatory frequency sweep test with a frequency range of 0.1 to 20 rad/s at a strain value of 2% (within the linear visco‐elastic region) was conducted. Following parameters were observed during the rheological study:

1. Storage modulus (Gꞌ), 2. Loss modulus (Gꞌꞌ), and 3. Shear Stress 4. Shear rate.

#### 2.3.6 Sensory evaluation

Sensory quality attributes such as color, flavor, taste, and overall acceptability of apple sauce were evaluated by semi‐trained panelists. The panelists were provided with a sample of apple sauce and were requested to assign scores based on color, flavor, taste, and overall acceptability using 5 point scale. Panelists were screened for oral lesions, specific anosmia, and cigarette use. A positive response to any of the questions resulted in exclusion. Each panelist tasted 10 apple sauce samples presented in a random order using an incomplete block design, which included the 2 treatments listed. The panelists were given a break of twenty minutes after each sample test. The overall scores were recorded in a blank A4 size sheet with a ball pen, and the mean was calculated.

#### 2.3.7 Statistical analysis

The data were analyzed by analysis of variance (ANOVA) using statistical software SPSS 16. A multiple comparison procedure of the means was performed by Post hoc test. The significance of the differences was defined at *p* ≤ .05.

## 3 RESULTS AND DISCUSSIONS

### 3.1 Total Soluble Solids (TSS) and Total Solids (TS)

From Table 2, it is clear that TSS and TS increased throughout the storage period of 45 days for refrigerated samples; however, increase in TSS is non‐significant. The samples stored at ambient temperature show an increase in TSS up to 30 days, and at the end of the storage period, there was a significant decrease in TSS and a similar trend was reported by Levent and Alpaslan (2018). The increase in TSS and TS can be explained by the fact that during the storage period breakdown of higher sugars into lower or simpler sugars takes place (Hussain et al., 2015). The TSS content of sauces was the lowest in ambient stored Chamures, and the TSS content of sauce was highest in refrigerated sample Mollies Delicious (C0) as shown in Table 2. The main factors affecting the TSS are storage duration (Hussein et al., 2020), chemical reactions, and temperature of storage that can change both pH and TSS of the stored product (Kaushik et al., 2014; Saleem et al., 2011). The increase in TS and TSS may also be due to hydrolysis of starch or other complex carbohydrates resulting in the formation of mono and disaccharides, and subsequently, a decline in these parameters is predictable as they are primary substrates for respiration (Aly et al., 1981; Wills et al., 1980).

**TABLE 2:** Physiochemical analysis of Apple sauce (TSS, total Sugars, pH and titratable acidity)

Cultivar	Refrigerated				Mean	Ambient				Mean
	0 day	15 day	30 day	45 day		0 day	15 day	30 day	45 day	
Change in TSS during storage										
American Apirouge	30.01 ± 0.1^aA^	30.03 ± 0.1^aA^	30.06 ± 0.02^bA^	30.11 ± 0.02^cA^	30.05	30.26 ± 0.25^bC^	30.5 ± 0.1^cB^	30.50 ± 0.35^cA^	28.6 ± 0.2^a C^	29.96
Delicious	30.20 ± 0.2^bC^	30.3 ± 0.1^cC^	30.5 ± 0.2^dB^	30.11 ± 0.02^aA^	30.28	30.16 ± 0.15^bA^	30.4 ± 0.2^cA^	30.50 ± 0.35b^dA^	28.6 ± 0.2^a C^	29.91
Red Delicious	30.10 ± 0.1^a B^	30.3 ± 0.1^bC^	30.6 ± 0.2^cC^	30.11 ± 0.02^aA^	30.28	30.26 ± 0.25^bC^	30.5 ± 0.2^cB^	30.7 ± 0.2^d B^	28.6 ± 0.2^a C^	30.01
Mollies Delicious	30.20 ± 0.2^bC^	30.3 ± 0.1^cC^	30.6 ± 0.2^dC^	30.11 ± 0.02^aA^	30.30	30.26 ± 0.25^bC^	30.5 ± 0.20^c B^	30.7 ± 0.2^d B^	27.6 ± 0.2^aB^	29.76
Chamure	30.10 ± 0.10^aB^	30.1 ± 0.20^aB^	30.7 ± 0.2^cD^	30.14 ± 0.02^bA^	30.26	30.21 ± 0.20^bB^	30.5 ± 0.20^c B^	30.7 ± 0.2^dB^	27.4 ± 0.2^a A^	29.70
Mean	30.12	30.21	30.49	30.12		30.23	30.48	30.62	28.16	
Change in Total sugars during storage										
American Apirouge	18.14 ± 0.0^aC^	18.26 ± 0.02^bC^	18.34 ± 0.02^cC^	18.44 ± 0.02^dC^	18.29	18.9 ± 0.02^dC^	18.30 ± 0.02^aC^	18.41 ± 0.02^bC^	18.51 ± 0.02^cC^	18.53
Delicious	20.14 ± 0.02^aE^	20.26 ± 0.02^bE^	20.36 ± 0.02^cE^	20.48 ± 0.02^dE^	20.31	20.16 ± 0.02^aE^	20.27 ± 0.02^bE^	20.38 ± 0.02^cE^	20.5 ± 0.02^dE^	20.33
Red Delicious	15.14 ± 0.02^aA^	15.18 ± 0.02^aA^	15.28 ± 0.02^bA^	15.38 ± 0.02^cA^	15.24	15.18 ± 0.01^aA^	15.28 ± 0.01^bA^	15.37 ± 0.02^cA^	15.38 ± 0.01^cA^	15.30
Mollies Delicious	19.70 ± 0.02^ad^	19.72 ± 0.02^aD^	19.84 ± 0.02^bD^	19.96 ± 0.02^cD^	19.80	19.74 ± 0.02^aD^	19.84 ± 0.02^bD^	19.97 ± 0.02^cD^	20.1 ± 0.02^dD^	19.91
Chamure	17.8 ± 0.02^aB^	17.92 ± 0.02^bB^	18.14 ± 0.02^cB^	18.26 ± 0.02^dB^	18.03	17.89 ± 0.02^aB^	17.98 ± 0.02^bB^	18.17 ± 0.02^cB^	18.28 ± 0.02^dB^	18.08
Mean	18.18	18.27	18.39	18.50		18.37	18.33	18.46	18.55	
Changes in pH during storage										
American Apirouge	4.05 ± 0.02^bA^	4.03 ± 0.02^bA^	4.01 ± 0.02^bB^	3.97 ± 0.02^aA^	4.01	4 ± 0.02^bA^	3.98 ± 0.02^bA^	3.94 ± 0.02^aA^	3.92 ± 0.02^aA^	3.96
Delicious	4.04 ± 0.02^cA^	4.02 ± 0.02^bA^	4.00 ± 0.02^bB^	3.97 ± 0.02^aA^	4.01	4.02 ± 0.02^bA^	4.00 ± 0.02^bA^	3.98 ± 0.02^abB^	3.96 ± 0.02^aB^	3.99
Red Delicious	4.04 ± 0.02^bA^	4.02 ± 0.02^bA^	3.98 ± 0.02^aA^	3.95 ± 0.02^aA^	3.99	4.01 ± 0.02^cA^	3.99 ± 0.02^bA^	3.97 ± 0.02^bB^	3.94 ± 0.03^aB^	3.98
Mollies Delicious	4.06 ± 0.02^cA^	4.04 ± 0.02^cA^	4.00 ± 0.02^bB^	3.96 ± 0.02^aA^	4.01	4.06 ± 0.02^cB^	4.04 ± 0.02^cB^	3.98 ± 0.02^bB^	3.95 ± 0.03^aB^	4.01
Chamure	4.07 ± 0.02^cA^	4.05 ± 0.02^cA^	4.01 ± 0.02^bB^	3.97 ± 0.02^aA^	4.02	4.06 ± 0.02^bB^	4.04 ± 0.02^bB^	4.03 ± 0.02^bC^	3.95 ± 0.02^aB^	4.02
Mean	4.05	4.03	4.00	3.96		4.03	4.01	3.98	3.94	
Changes in titratable acidity during storage										
American Apirouge	4.27 ± 0.02^aD^	4.27 ± 0.02^aD^	4.31 ± 0.02^bE^	4.35 ± 0.02^cD^	4.30	4.58 ± 0.02^aC^	4.60 ± 0.02^a D^	4.64 ± 0.02^bD^	4.68 ± 0.02^cD^	4.62
Delicious	3.58 ± 0.02^aB^	3.60 ± 0.02^aB^	3.64 ± 0.02^bB^	3.68 ± 0.02^cB^	3.62	3.70 ± 0.02^aA^	4.06 ± 0.02^bB^	4.10 ± 0.02^cB^	4.13 ± 0.02^cB^	3.99
Red Delicious	3.31 ± 0.03^aA^	3.34 ± 0.03^aA^	3.39 ± 0.02^bA^	3.43 ± 0.02^cA^	3.37	3.57 ± 0.02^aA^	3.59 ± 0.02^aA^	3.63 ± 0.02^bA^	3.67 ± 0.02^cA^	3.615
Mollies Delicious	4.05 ± 0.02^aC^	4.07 ± 0.02^aC^	4.14 ± 0.02^bD^	4.17 ± 0.02^cC^	4.11	4.11 ± 0.03^a B^	4.14 ± 0.03^aC^	4.19 ± 0.02^bC^	4.23 ± 0.02^cC^	4.17
Chamure	4.02 ± 0.02^aC^	4.04 ± 0.02^aC^	4.08 ± 0.02^bC^	4.14 ± 0.02^cC^	4.07	4.11 ± 0.03^a B^	4.14 ± 0.03^aC^	4.19 ± 0.02^bC^	4.25 ± 0.02^cC^	4.17
Mean	3.846	3.864	3.912	3.954		4.01	4.11	4.15	4.19	

Note

Values are mean ± standard deviation (*n* = 3).

a‐b: Within a row, different letters indicate significant differences among the storage period (*p* <.05).

A‐B: Within a column, different letters indicate significant differences among different cultivars of apple (*p* <.05).

### 3.2 pH

It is evident from Table 2 that the pH showed a decreasing trend while titratable acidity increases with the advancement in storage periods. American Apirouge cultivar showed the highest acidity at both refrigerated and ambient conditions. The difference in titratable acidity for different apple cultivar sauces was very little as the percentage of acetic acid ranges between 3.3 and 4.6. There were little differences in pH in all cultivars. However, with the advancement of the storage period (30 days) difference in pH became significant for red delicious with respect to all other cultivars. pH values and titratable acidity values show a slight change during first 30 days of storage, however, change in titratable acidity and pH became significant at 45 days of storage period for all the cultivars with respect to zero days of storage. A slight increase in titratable acidity with the advancement of the storage period was also observed by (Touati et al., 2014; Levent and Alpaslan, 2018). The overall increase in titratable acidity was more for ambient samples as compared to refrigerated samples for all the cultivars. The observation resembles the study of (Wisal et al., 2013) for strawberry juice. The increase in acidity can be explained by the fact that with the advancement of the storage period enzymes can catalyze the breakdown of sugars into acids and hence can increase the acidity (Kumhar et al., 2014).

### 3.3 Color

As is evident from Table 3 the values for lightness (L*), decreases slightly for 45 days of storage at refrigerated and ambient conditions. “L*” values of sauce show slight change during the storage period as the sauce becomes darker, which corresponds to the decrease in “L” value (Wickramarachchi & Ranamukhaarachchi, 2005). The highest decrease in the “L” value has been observed in samples D0 and D1 (Chamure cultivar). The a* and b* values did not show any significant differences and increased slightly with storage time. The decrease in the L* value and increase in a* and b* values can be related to the increase in reducing sugars and amino groups because of higher temperatures during processing and similar findings were reported by (Sunthanont, 1998; Gonzalez‐Buesa et al., 2011; Schweiggert et al., 2011).

**TABLE 3:** Color parameter of apple sauce during storage (Color analysis)

Cultivar	Refrigerated				Mean	Ambient				Mean
	0 day	15 day	30 day	45 day		0 day	15 day	30 day	45 day	
Changes in “L*” value's during storage										
American Apirouge	31.38 ± 0.53^dA^	30.84 ± 0.02^cD^	30.74 ± 0.02^bD^	29.93 ± 0.02^aD^	30.72	32.08 ± 0.02^dB^	31.97 ± 0.01^cE^	30.04 ± 0.02^bD^	28.12 ± 0.04^aD^	30.55
Delicious	32.08 ± 0.03^dC^	30.16 ± 0.02^cB^	28.05 ± 0.02^bA^	27.95 ± 0.03^aC^	29.56	32.08 ± 0.01^dB^	30.64 ± 0.02^cD^	28.60 ± 0.02^bC^	27.73 ± 0.02^aC^	29.76
Red Delicious	32.08 ± 0.01^dC^	30.09 ± 0.02^cA^	28.14 ± 0.02^bB^	27.32 ± 0.03^aA^	29.41	32.08 ± 0.02^dB^	30.04 ± 0.02^cA^	28.05 ± 0.02^bA^	27.52 ± 0.02^aB^	29.42
Mollies Delicious	32.01 ± 0.02^dB^	30.60 ± 0.02^cC^	28.44 ± 0.02^bC^	27.32 ± 0.02^aA^	29.59	31.99 ± 0.01^dA^	30.09 ± 0.02^cB^	28.60 ± 0.02^bC^	27.75 ± 0.01^aC^	29.61
Chamure	32.04 ± 0.02^dB^	30.16 ± 0.02^cB^	28.15 ± 0.02^bB^	27.46 ± 0.02^aB^	29.45	31.98 ± 0.01^dA^	30.14 ± 0.02^cC^	28.11 ± 0.02^bB^	27.26 ± 0.02^aA^	29.37
Mean	31.92	30.37	28.70	27.99		32.04	30.57	28.68	27.67	
Changes in “a*” value's during storage										
American Apirouge	24.14 ± 0.02^aB^	24.12 ± 0.01^aA^	24.12 ± 0.02^aA^	24.14 ± 0.02^aA A^	24.13	24.10 ± 0.02^aA^	24.1 ± 0.02^aA A^	24.11 ± 0.02^aA^	24.15 ± 0.02^bAB^	24.12
Delicious	24.14 ± 0.01^aB^	24.13 ± 0.02^aA^	24.16 ± 0.01^bA^	24.21 ± 0.02^cB^	24.16	24.11 ± 0.01^aA^	24.18 ± 0.01^bB^	24.1 ± 0.02^aA^	24.1 ± 0.02^aBA^	24.12
Red Delicious	24.15 ± 0.0^bB^	24.21 ± 0.02^cD^	24.15 ± 0.02^bA^	24.11 ± 0.01^aCA^	24.15	24.14 ± 0.02^aB^	24.15 ± 0.02^aB^	24.26 ± 0.02^bC^	24.29 ± 0.01^cD^	24.21
Mollies Delicious	24.16 ± 0.01^aB^	24.18 ± 0.01^aB^	24.23 ± 0.02^bC^	24.26 ± 0.02^cC^	24.21	24.13 ± 0.02^aAB^	24.16 ± 0.02^bB^	24.23 ± 0.02^cB^	24.26 ± 0.02^dC^	24.19
Chamure	24.07 ± 0.02^aA^	24.15 ± 0.02^bC^	24.18 ± 0.02^cB^	24.24 ± 0.02^dC^	24.16	24.15 ± 0.02^aB^	24.18 ± 0.02^bB^	24.21 ± 0.02^cB^	24.24 ± 0.02^dC^	24.19
Mean	24.13	24.16	24.17	24.19		24.13	24.15	24.18	24.21	
Changes in “b*” value's during storage										
American Apirouge	42.05 ± 0.02^aA^	42.06 ± 0.02^aA^	42.08 ± 0.01^aA^	42.07 ± 0.01^aA^	42.06	42.06 ± 0.02^aA^	42.08 ± 0.02^aA A^	42.14 ± 0.02^bB^	42.13 ± 0.02^bA^	42.10
Delicious	42.08 ± 0.02^bA^	42.15 ± 0.02^cB^	42.11 ± 0.02^aB^	42.16 ± 0.01^cB^	42.12	42.14 ± 0.02^bB^	42.16 ± 0.02^bC^	42.06 ± 0.02^aB A^	42.15 ± 0.02^aA^	42.12
Red Delicious	42.17 ± 0.01^cC^	42.14 ± 0.02^bB^	42.08 ± 0.02^aA^	42.13 ± 0.02^bC^	42.13	42.14 ± 0.02^aB^	42.22 ± 0.01^bD^	42.26 ± 0.02^cD^	42.25 ± 0.02^cB^	42.22
Mollies Delicious	42.10 ± 0.02^aB^	42.16 ± 0.02^bB^	42.23 ± 0.02^cC^	42.26 ± 0.02^dD^	42.19	42.16 ± 0.01^aB^	42.18 ± 0.02^aC^	42.20 ± 0.01^bC^	42.25 ± 0.02^cB^	42.19
Chamure	42.16 ± 0.02^aC^	42.19 ± 0.02^bB^	42.22 ± 0.02^cC^	42.25 ± 0.0^dD^	42.20	42.05 ± 0.03^aA^	42.13 ± 0.02^bB^	42.2 ± 0.02^c C^	42.23 ± 0.02^cB^	42.15
Mean	42.11	42.14	42.14	42.17		42.11	42.15	42.17	42.20	

Note

Values are mean ± standard deviation (*n* = 3).

a‐b: Within a row, different letters indicate significant differences among the storage period (*p* <.05).

A‐B: Within a column, different letters indicate significant differences among different cultivars of apple (*p* <.05).

### 3.4 Sensory evaluation

Overall acceptability (OA) for Molis and Chamure was highest as compared to other cultivars as shown in Table 4. The increase in overall acceptability can be correlated with an increase in TSS and increase in titratable acidity, highest OA value 4.314 and 4.33 was observed for Mollies Delicious and Chamure cultivar, respectively (Kumhar et al., 2014; Vidhya & Narain, 2011).

**TABLE 4:** Sensory analysis of Zero‐day and 45‐days sample

Cultivar	Refrigerated				Overall acceptability	Mean	Ambient				Overall acceptability	Mean
	Color	Taste	Aroma	Appearance		Mean	COLOR	Taste	Aroma	Appearance		
Zero‐day analysis												
American Apirouge	4.25 ± 0.02	4.04 ± 0.02	4.36 ± 0.02	4.04 ± 0.01	4.34 ± 0.02	4.21	4.23 ± 0.02	4.02 ± 0.02	4.34 ± 0.02	4.02 ± 0.02	4.32 ± 0.02	4.19
Delicious	4.26 ± 0.03	4.65 ± 0.02	4.36 ± 0.02	4.22 ± 0.02	4.44 ± 0.02	4.39	4.21 ± 0.01	4.63 ± 0.02	4.34 ± 0.02	4.18 ± 0.02	4.40 ± 0.02	4.35
Red Delicious	4.20 ± 0.02	4.34 ± 0.02	4.35 ± 0.01	4.25 ± 0.02	4.26 ± 0.02	4.28	4.16 ± 0.02	4.30 ± 0.02	4.33 ± 0.02	4.23 ± 0.02	4.22 ± 0.02	4.25
Mollies Delicious	4.22 ± 0.02	4.40 ± 0.02	4.34 ± 0.02	4.23 ± 0.02	4.38 ± 0.02	4.31	4.18 ± 0.02	4.34 ± 0.02	4.32 ± 0.02	4.22 ± 0.02	4.36 ± 0.02	4.28
Chamure	4.24 ± 0.02	4.42 ± 0.02	4.33 ± 0.02	4.26 ± 0.02	4.40 ± 0.02	4.33	4.20 ± 0.02	4.40 ± 0.02	4.30 ± 0.02	4.22 ± 0.02	4.34 ± 0.02	4.29
Mean	4.23	4.37	4.35	4.20	4.36		4.20	4.34	4.33	4.17	4.33	
45 days analysis												
American Apirouge	4.07 ± 0.02	3.84 ± 0.02	4.19 ± 0.02	3.86 ± 0.02	3.98 ± 0.02	3.988	4.05 ± 0.02	3.82 ± 0.02	4.17 ± 0.02	3.82 ± 0.02	3.94 ± 0.02	3.96
Delicious	4.16 ± 0.03	4.44 ± 0.02	4.28 ± 0.02	4.30 ± 0.02	4.28 ± 0.02	4.292	4.21 ± 0.01	4.63 ± 0.02	4.34 ± 0.02	4.18 ± 0.02	4.40 ± 0.02	4.35
Red Delicious	4.06 ± 0.02	4.44 ± 0.02	4.04 ± 0.01	3.94 ± 0.02	4.16 ± 0.02	4.128	3.93 ± 0.02	4.08 ± 0.02	4.10 ± 0.02	3.95 ± 0.02	3.96 ± 0.02	4.00
Mollies Delicious	3.86 ± 0.02	3.00 ± 0.02	4.03 ± 0.02	3.93 ± 0.02	3.94 ± 0.04	3.752	3.92 ± 0.02	4.10 ± 0.02	4.04 ± 0.02	3.93 ± 0.02	4.08 ± 0.02	4.01
Chamure	3.88 ± 0.02	4.04 ± 0.02	4.02 ± 0.02	3.92 ± 0.02	4.08 ± 0.02	3.988	3.90 ± 0.02	4.10 ± 0.02	4.00 ± 0.02	3.92 ± 0.02	4.04 ± 0.02	3.99
Mean	4.01	3.93	4.11	3.99	4.09		4.00	4.15	4.13	3.96	4.08	

### 3.5 Rheological evaluation

#### 3.5.1 Frequency sweep

**FIGURE 1:**
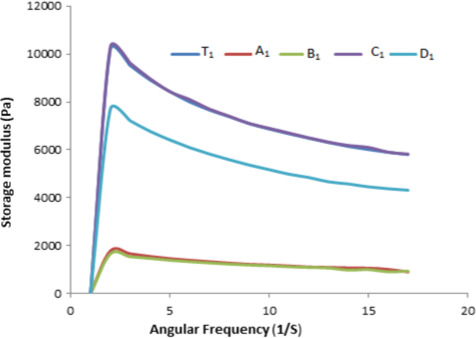
Zero‐day analysis frequency versus Storage modulus

**FIGURE 2:**
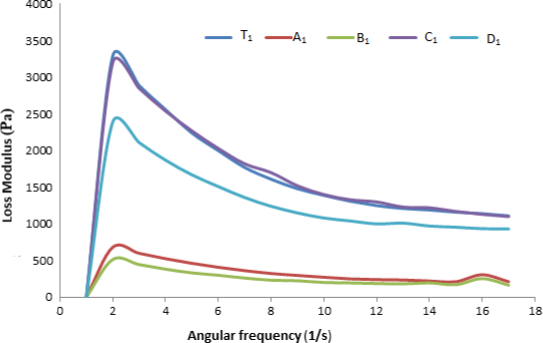
Zero‐day analysis frequency versus Loss modulus

**FIGURE 3:**
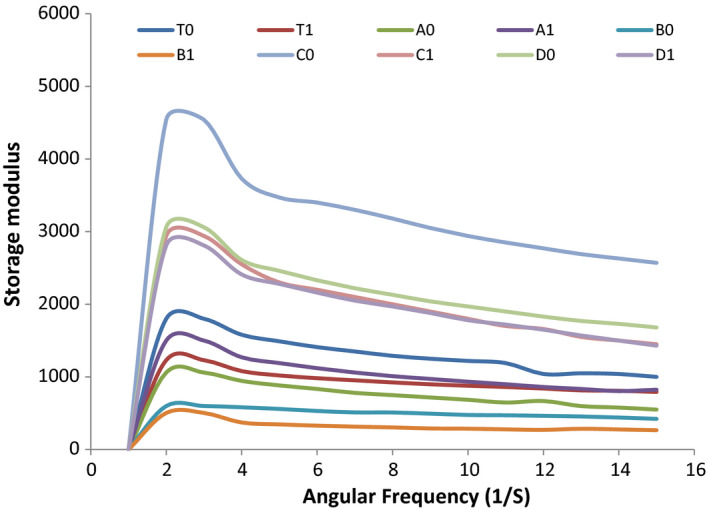
Forty‐five (45) day analyzed of angular frequency versus storage modulus

**FIGURE 4:**
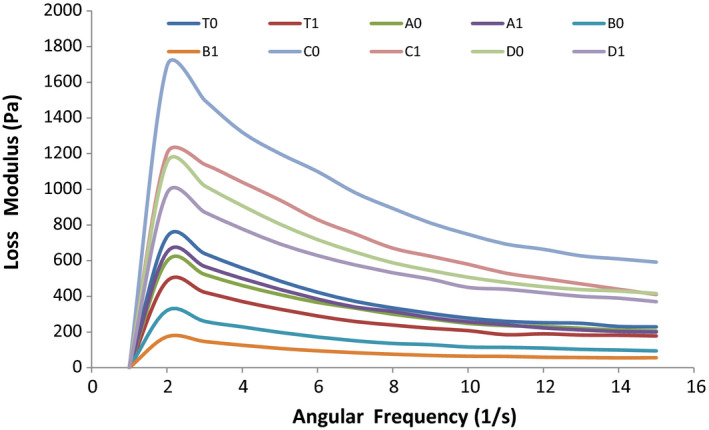
Forty‐five (45)‐day analysis of angular frequency versus Loss modulus

A frequency sweep was performed within the viscoelastic region. The mechanical spectra obtained from frequency sweep tests for apple sauces are shown in Figures 1, 2, 3, and 4. These rheological properties are measurements of the elastic modulus (Gʹ) and the viscous modulus (G″) of foods when stress is applied to them. As is represented by the figures, Gʹ is greater than G″ for almost all the samples that are indicative of the apple sauce being more elastic as compared to the viscous nature. Similar observations were noted for Apricot sauce(Levent and Alpaslan, 2018; Augusto et al., 2012) Jabuticaba pulp by (Sato and Cunha, 2009; Tanon et al., 2009) and sorbitol cherry jam (Bakshi et al., 2020). The graphical representation Gʹ and G″ for the samples A1, D1 shows a greater slope for Gʹ and G″, while C1 and B1 show a lower slope. With the advancement of the storage period, there is a change in Gʹ and G″ and the change can be associated with the changes in pH and TSS during storage. The highest decrease in Gʹ and G″ has been observed in C1 and C2 samples because these show the highest change in pH during storage. The ambient storage samples show a high change in rheological parameters than refrigerated stored samples. The reason might be that at ambient storage change in chemical parameters is more as compared to refrigerated storage which in turn affects the rheological parameters. The study resembles the study of Guerrero and Katlijn (Guerrero and Alzamora, 1998; Katlijn et al., 2013).

#### 3.5.2 Shear rate versus viscosity

**FIGURE 5:**
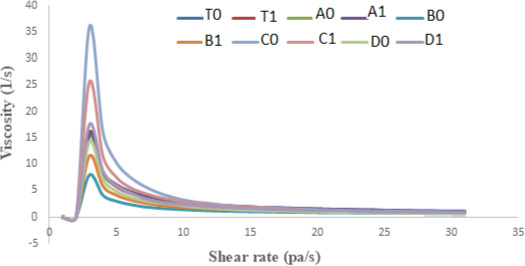
Zero‐day analysis of apparent viscosity versus Shear rate

**FIGURE 6:**
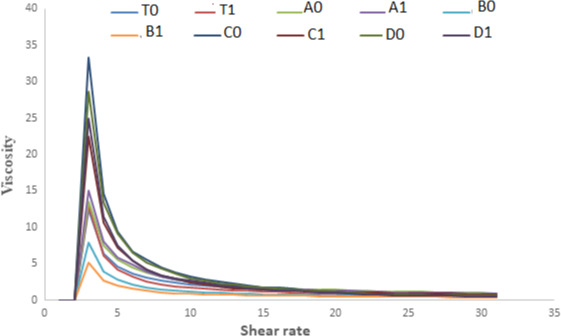
Apparent viscosity of both refrigerated and ambient stored samples at the end of storage (45 days)

From Figures 5 and 6, it is clear that with the increase in the shear rate the apparent viscosity decreases and the decrease in viscosity shows linear relation with the shear rate which indicates the shear‐thinning behavior of apple sauce. The reason behind the decrease in viscosity can be attributed to the fact that heating during the sauce preparation can modify cellular structure especially cell wall structure, which can result in the softening of the pectin and thus changing the rigidity of the cells. Redgwell (Redgwell et al., 2008) and Abd‐Elhady (Abd‐Elhady, 2014) in their studies on apple pulp and strawberry, respectively, reported that the decrease in viscosity can be explained by the fact that during the preparation of sauces heating can destabilize the cellulose network, which results in the decrease in viscosity of apple samples. The sample of apple sauce with the highest Brix showed the lowest consistency. A similar study was reported by Ditchfield et al. (2004). At the end of storage (Figure 4), the sample shows a gradual decrease in a rheological value. The highest decrease has been observed in C_1_ and C_0_. The decrease in the rheological behavior can be because of the structural breakdown of molecules that occurs initially during the preparation of the samples due to the generation of the different operating forces and also because of the increased alignment of constituted molecules in the later stages. In all the samples, the rate of the structural breakdown was higher in the initial phase and the decrease was marginal in later stages (Nindo et al., 2007).

## 4 CONCLUSION

The results of our study indicated that the apple sauces stored at refrigerated conditions prompted less physiochemical changes as compared to the storage at a temperature of 25°C (ambient conditions). Interaction of storage time and the temperature had a significant effect on the stability of apple sauce. An increase in acidity has been observed in both cases, while ambient storage shows the highest increase than that of refrigerated storage. the highest acidity has been found in the D_0_ sample while the lowest acidity has been observed in B_0_. Sensory analysis of sauces has shown A0 as the highest acceptable product. Frequency sweep tests demonstrated that the elastic modulus was greater than the viscous modulus for all the samples, and both the moduli decreased with a decrease in frequency. Viscosity showed a linear decrease with the increasing shear stress. The knowledge provided by the study can be used for the development of sauces from different cultivars of apple in terms of storage behavior and consistency for industrial applications.

## CONFLICT OF INTEREST

The authors declare that there are no conflicts of interest regarding the publication of this paper.

## ETHICAL APPROVAL

This study did not involve any animal or human testing.
